# Methodological Guidelines for Systematic Assessments of Health Care Websites Using Web Analytics: Tutorial

**DOI:** 10.2196/28291

**Published:** 2022-04-15

**Authors:** Edwin Lauritz Fundingsland Jr, Joseph Fike, Joshua Calvano, Jeffrey Beach, Deborah Lai, Shuhan He

**Affiliations:** 1 Department of Emergency Medicine Homer Stryker MD School of Medicine Western Michigan University Kalamazoo, MI United States; 2 Department of Physical Medicine and Rehabilitation Medical Center University of Nebraska Omaha, NE United States; 3 Department of Anesthesiology and Critical Care University of New Mexico Hospital Albuquerque, NM United States; 4 College of Osteopathic Medicine Rocky Vista University Parker, CO United States; 5 Division of Psychology and Language Sciences University College London London United Kingdom; 6 Center for Innovation in Digital HealthCare Lab of Computer Science Massachusetts General Hospital Boston, MA United States

**Keywords:** Google Analytics, website usability, conversion rate, website engagement, user demographics, website traffic, website content, internet browsers, healthcare websites, web analytics, healthcare industry, usability

## Abstract

With the growing importance of communicating with the public via the web, many industries have used web analytics to provide information that organizations can use to better achieve their goals. Although the importance of health care websites has also grown, the health care industry has been slower to adopt the use of web analytics. Web analytics are the measurement, collection, analysis, and reporting of internet data used to measure direct user interaction. Our objective is to provide generalized methods for using web analytics as key performance metrics to evaluate websites and outline actionable recommendations for improvement. By deconstructing web analytic categories such as engagement, users, acquisition, content, and platform, we describe how web analytics are used to evaluate websites and how improvements can be made using this information. *Engagement* is how a user interacts with a website. It can be evaluated using the daily active users to monthly active users (DAU/MAU) ratio, bounce rate, pages viewed, and time on site. Poor engagement indicates potential problems with website usability. *Users* pertains to demographic information regarding the users interacting with a website. This data can help administrators understand who is engaging with their website. *Acquisition* refers to the overall website traffic and the method of traffic, which allows administrators to see how people are accessing their website. This information helps websites expand their methods of attracting users. *Content* refers to the overall relevancy, accuracy, and trustworthiness of a website’s content. If a website has poor content, it will likely experience difficulty with user engagement. Finally, *platform *refers to the technical aspects of how people access a website. It includes both the internet browsers and devices used. By providing detailed descriptions of these categories, we have identified how web administrators can use web analytics to systematically assess their websites. We have also provided generalized recommendations for actionable improvements. By introducing the potential of web analytics to augment usability and the conversion rate, we hope to assist health care organizations in better communicating with the public and therefore accomplishing the goals of their websites.

## Introduction

### Background

With the continually growing global importance of the World Wide Web, websites have become a crucial communication channel for corporations, political groups, and organizations because of their capability to rapidly disseminate information to various audiences at a low cost [1]. Web analytics has become a mainstay of commercial industries and even a commercial industry itself. The web analytics market was valued at US $2.63 billion in 2018 and is projected to reach US $10.73 billion by 2026, growing at a compound annual growth rate of 19.3% from 2019 to 2026 [2]. The field of medicine, however, remains hindered as stakeholders in health care have been slow to adopt digital innovations. Studies have shown that the adoption of digital technologies can improve the performance of health care processes, increase efficiency, and enable the delivery of higher-quality care and reduced response times, with many benefits for several stakeholders, such as national health systems, clinicians, and patients [3]. For organizations to achieve their goals in use and impact, their website’s communication capacity is key. A website that cannot effectively communicate is not serving its purpose. Communication capacity can be measured through the usability and conversion rate of a website [1].

Studies have shown a relationship between the usability of health care websites and the credibility ascribed by its users [4]. The International Organization for Standardization defines usability as “the extent to which a system, product or service can be used by specific users to achieve specified goals with effectiveness, efficiency and satisfaction in a specified context of use” [5]. Measures of effectiveness, efficiency, and satisfaction can be viewed as key web analytic metrics and if optimized, can lead to increased website success. By augmenting usability, a website can reach a higher level of engagement and achieve its desired objectives. A lack of design errors, following established design conventions, and ease of navigation are important features emphasized in the literature [6]. When users have difficulty accessing or using a website, they are likely to move on to another resource, while a website that uses usability metrics is more likely to retain users.

Other industries have established user expectations for their respective websites; health care websites are facing the need to conform [7,8]. Studies have been conducted which evaluate usability in areas such as e-commerce, e-government, mobile news apps, and library websites [9-12]. More recently, there have been increasing usability studies focusing on websites within the health care sector, such as websites for emergency medicine residency programs, digital health care centers, hospitals, and cancer centers [13-16]. With the growing importance of website usability and the conversion rate in the health care sector, web analytics can provide health care stakeholders with an easily accessible tool to assist their evaluation of usability and measure conversion rate.

The conversion rate is closely intertwined with usability. It measures the number of users who perform the desired goal of the page (ie, buying a product or filling out a form) relative to the total users [17]. A high conversion rate separates a successful website from an unsuccessful website. 

Web analytics refers to the collection, analysis, and reporting of internet data for the purposes of understanding and optimizing web use [18]. On-site web analytics are used to measure direct user interaction, such as the number of visitors, time spent on a website, and click path [19]. Overall, web analytics can contribute to determining a website’s usability and conversion rates [1]. This collection of data is used to analyze the performance of a site and can allow websites to improve their persuasion and relevance [20]. It is important to note, however, that web analytics do not provide a comprehensive measurement of website usability. Measurement of usability consists of additional variables that are outside the scope of this publication.

To the authors’ knowledge, there is no recent paper outlining how web analytics can be applied broadly to the field of health care. By evaluating the categories of engagement, users, acquisition, content, and platform, we aim to create a universal framework of web analytics that can be applied to health care websites to improve the quality and effectiveness of these websites. 

### Objectives

This aim of this tutorial is to (1) provide a basic understanding of definitions and methods pertaining to web analytics, (2) create a framework for using web analytics to evaluate the effectiveness of health care websites specifically, and (3) outline the actionable implications of web analytics to assist health care websites in achieving their goals.

## Methods

### Google Analytics

Google Analytics (GA) is a web analytics service that has been offered by Google since 2005. It is the most widely used web analytics tool, with 84.1% of the market share [21]. It can be used for both websites and apps, across iOS and Android devices. As of August 2013, GA was reportedly used by 66.2% of the 10,000 most popular websites [22]. GA offers a free version that can be used by those with a graphical user interface and without software engineering skills. Any owner of a website or app can sign up for a GA account. 

### Engagement 

An engagement analysis evaluates user activity and is one of the most used analytic tools. It describes how users interact with websites [19]. Factors that are often addressed include how often visitors return to the site, how often new visitors become returning visitors, pages visited per session, and duration of visits [19].

#### Daily Active User to Monthly Active User (DAU/MAU) Ratio

When evaluating overall engagement, *1-day active users* refers to users who have been active at least once in the previous day, *7-day active users* refers to users who have been active at least once in the previous 7 days, and so on for 14- and 28-day active users. 1-day active users are referred to as daily active users (DAU) and 28-day active users are referred to as monthly active users (MAU). The ratio of DAUs to MAUs, DAU/MAU, can be expressed as a percentage to understand user engagement; this measure was first popularized by Facebook and has since become a popular key performance indicator (KPI), with some venture capitalists considering a ratio of over 20% favorable and over 50% excellent [23]. Assessing the DAU/MAU ratio can indicate whether a site is attracting users at its intended or expected frequency. For instance, a rideshare website may expect to see a high DAU/MAU ratio. On the other hand, a flight booking site may see a lower DAU/MAU ratio. Regarding health care websites, many sites have goals of continued user involvement. A low DAU/MAU ratio can be used as an indication that the relevancy of the content and usability of a website can be improved. It is important to view this metric in the context of a website’s desired goals. For health care websites that do not desire continued engagement, this may not be a relevant metric and therefore would not correlate with usability issues.

#### Bounce Rate

The *bounce rate* is another metric of engagement referring to the percentage of single-session users (ie, users who visit the site and “bounce” without interacting further, as opposed to users who interact with at least 1 additional page). A session is recorded by GA each time a user visits the site, beginning as soon as the site is first loaded and ending after 30 minutes of inactivity. Using this metric, the website host is provided with insight into the user’s engagement with their product. Navigating to other pages of a website or application is typically viewed as an active event triggered by the user. Similar to the DAU/MAU ratio, the bounce rate indicates users who are not achieving the desired interaction with the website. A high bounce rate may suggest a usability issue steering users away from the page. More than simply content, many things can cause users to avoid visiting additional pages. One example is slow loading speeds. If a website is loading too slowly, users may leave the site before viewing any of the content. According to a recent Google study, a website that takes longer than 3 seconds to load on a mobile device loses approximately 53% of its users and the average mobile website speed is around 18 seconds [24]. This issue can be addressed simply by reducing conflicting technology on the back-end server [13,14]. As with the DAU/MAU ratio, web administrators must view this metric in the proper context. If they do not desire continual engagement within a single session, the bounce rate is not a useful metric.

#### Page Views

The number of pages per session is the number of pages within the site that a user visits during a single session and indicates how thoroughly a user is engaging with a website. A page view is counted every time a website is loaded, and this can be tracked using GA [1]. Similar to the bounce rate, if users are accessing a website but not interacting with additional pages, there may be an issue with its usability. The goal of many websites is for people to view subpages with additional content but various issues could interrupt this, one being front-end web page design. If users are not easily finding links to subpages, they may lose interest and bounce. By working with marketing specialists, web administrators can improve their webpage design and ease user navigation. As with the bounce rate, if continual engagement within a session is not a desired outcome, this metric is not a helpful measure of engagement.

#### Time on Site

As the name suggests, *time on site* refers to the duration of time a user spends on a website. If the same visitor comes back several hours later or the next day, a new session is counted. This is considered a key indicator of how successfully a website is engaging visitors. It has been suggested that time on site is an indication of website usability. However, this is operating under the notion that the greater the usability of a website, the more time a user will spend on it. A long session duration may suggest that users are spending more time reviewing the detail of a website’s content, while a short duration may suggest poor usability. It is important to analyze time on site and page views together to dissect whether users are spending increased time on the site due to difficulty navigating it [1].

### Users 

Analytics can help health care centers understand who the users of their websites are. GA provides limited demographic information about users, including age and gender distribution and location. If a website has a target audience, they can monitor if they are reaching that demographic. If they are targeting a diverse population of users, they can also use these demographics to monitor their success. Using this information, web creators can better focus their efforts on the population viewing their website or target those who are not using the site. For example, if it is discovered that males over the age of 65 years are primarily accessing a men’s health website, the web administrators would know they are reaching part of their target demographic. However, they may want to make efforts in marketing to younger users as well.

### Acquisition

By employing use data to understand consumer needs, websites can increase their user acquisition [19]. *Acquisition* refers to the amount of traffic a website receives. *Sources* refer to the origins of a user’s traffic to the site. If the overall user volume of a website is low, the method of traffic can be an important variable to address to reach more users. By using acquisition data, administrators can see where there is room for improvement in reaching potential users.

#### Direct Traffic

*Direct traffic* refers to visitors who arrive on the site directly by typing the URL into the browser address bar, clicking on a bookmark, or clicking on a link in an email, text message, or chat. Direct traffic can be a strong indicator of brand strength as well as success in email, text message, and offline marketing. If a website is experiencing low volumes of direct traffic, they can increase their efforts in these forms of marketing and in improving their overall brand strength.

#### Referral Traffic

*Referral traffic* refers to visitors who arrived at the site via another website. This occurs when outside websites contain links to a given site. If referral traffic is low, websites can place more emphasis on promotion via other websites. For example, if an organization has multiple websites under their umbrella, they can use their content on one website to direct users to their other. Referral traffic also encourages organizations to increase website partnerships that mutually benefit both parties.

#### Organic Traffic

*Organic traffic* refers to visitors who arrived at the site via a search result page (eg, Google or Bing) and can be an indicator of strong content or search engine optimization (SEO). SEO is a method for increasing organic traffic that has gained popularity in many industries. Many users find websites by simply entering keywords into a search engine and choosing the website that seems most appropriate. By strategically strengthening a website’s content, web administrators can help move their website closer to the top of a search engine results page (SERP) and therefore increase traffic. The nuances of SEO are outside the scope of this publication.

#### Social Traffic

*Social traffic* is similar to referral traffic, but it refers to traffic from social media platforms as opposed to traffic from other websites. Websites that are receiving low levels of social traffic can seek to implement, improve, and promote their social media presence on platforms such as Facebook, Twitter, and Instagram. Improved social media presence can also improve overall brand awareness.

### Content

Assessments of a website’s content can refer to the relevancy of information, the quality of multimedia content, and even grammar and spelling [13,14]. One of the most obvious reasons a website may not achieve its goals is its content not meeting the needs of users.

#### Relevancy

Concerning relevancy, the following questions should be posed: is a website’s information up to date and fact-driven, and does it provide answers people are seeking [13,14]? If the answer to any of these questions is no, users will not engage with a website. If a site is concerned with relevance, a solution may be to increase the frequency of content updates to ensure the information provided is not out of date. Additionally, it is particularly important for health care–related sites to have accurate and fact-driven content. Especially pertaining to health-related information, users will not engage with a website they believe to contain inaccurate information. 

#### Multimedia Content

Multimedia content can be evaluated by quantity and quality of resolution [13,14]. Seeking to further augment their content, websites can use multimedia to make their content more dynamic. Increasing the quality and quantity of videos, graphics, and animations has been shown to increase user engagement [13,14].

#### Spelling and Grammar

Spelling and grammar are important aspects of content quality. Even if a website’s content is up to date and accurate, users still may not trust it if there are obvious spelling and grammatical errors. There are easily accessible spelling and grammar tools available for websites to avoid this issue.

### Platform

To better understand potential areas of improvement for a website, engagement can be evaluated on each page. One can also assess the different browsers and devices through which users access a website to identify technical areas of improvement. 

#### Browser

A *browser* is the software application used to access the internet. Common browsers include Google Chrome (Google LLC), Internet Explorer (Microsoft Corp), and Safari (Apple Inc). If a website is not easily accessible on all major internet browsers, web administrators are automatically eliminating potential users. We have already discussed the benefits of SEO to improve the placement of a site on a SERP, but the improvement discussed here is made from a technological perspective. For example, if a website uses Java, a Google Chrome browser will not be able to support it [25]. It is wise for web developers to tailor their websites to the browsers of their users. GA data can provide information about which browsers are being used to access the website.

#### Device

Users are accessing websites on various devices, namely desktop computers or laptops, mobile phones, and tablets. Similar to browsers, if a website cannot be accessed on all devices, this eliminates an entire category of potential users. Tablets and smartphones are more commonly being used to access the internet; therefore, it is important that websites are mobile friendly. Administrators have the option to make separate mobile websites, but with mobile devices becoming more sophisticated, new methods have developed. One new and simpler method known as responsive design allows for the creation of one web page, then uses multiple sets of CSS rules to adjust formatting of the website to fit the size of the browser window [26].

## Discussion

### Main Recommendations

By providing detailed descriptions of categories such as engagement, users, acquisition, content, and platform, we have identified how web administrators can use web analytics to systematically assess their websites using tools such as GA. We have also provided generalized recommendations for actionable improvements that can be made to address website weaknesses. 

Although web analytics may be at an infant stage in the world of health care, it is very prevalent in other industries. By introducing the potential benefits of web analytics in the health care sector, we hope to continue the standardization of web practices that users have become accustomed to. Using web analytic tools in the proper context, health care website administrators can gain more information on user engagement and use this information to make improvements.

With the health care industry being slow to adapt to standards for website usability, we hope that the outlined methods and recommendations for using web analytics can be directed toward areas in need of improvement and increase the websites’ conversion rates. These recommendations can make a significant impact for health care organizations because they are actionable at a low cost. The potential of a website to improve persuasion and relevance has been established and by using web analytics, web administrators can easily expand upon this potential with a smaller financial burden compared to other methods.

### Limitations

The approaches outlined in this paper are intended to be broadly generalizable to health care–related websites such that they can be used by a wide spectrum of web administrators in the health care industry. However, each organization should tailor this approach to their unique objectives and considerations. This content serves primarily as an introduction to the potential benefits and methods of using web analytics, and future studies may focus on more specific use cases, such as applications for subfields in health care. 

Key web analytic metrics are not a comprehensive method for evaluating website usability. In certain cases, a degree of inference must be made to use web analytics as a reflection of a website’s usability. For example, the conversion rate can be used as a measure of a website’s effectiveness. However, if those viewing a website are not its targeted users, a poor conversion rate does not necessarily reflect poor usability. This underscores the importance of using various web analytic measures to gain a comprehensive perspective of user interaction. In the given scenario, administrators could examine the demographic characteristics of their websites users to determine if there is in fact an issue with usability. Similarly, metrics like the DAU/MAU ratio, bounce rate, and page views are used as a measure of website engagement, but it remains important to consider these measures within the context of a website’s targeted users and objectives. If it is not a website’s goal to promote continual access, the DAU/MAU ratio is not a useful measure for usability. Similarly, if it is not a website’s goal to foster continual engagement within each session, page views and the bounce rate are not useful.

Finally, these metrics are only one aspect of the overall capabilities of website usability analysis. Other methods to evaluate usability include user interviews and on-page heat mapping. Future studies delving into these methods would help improve our understanding of website usability in health care–related websites.

### Conclusions

Websites continue to be a primary method by which health care organizations interact with their consumers; however, the health care sector lags behind many other industries in using accepted and standardized website usability practices. With evidence pointing to the efficacy of using web analytics to augment the usability and conversion rate, health care organizations can benefit from adopting these practices to better accomplish the goals of their websites.

## Abbreviations

DAU: daily active users
DAU/MAU: daily active users to monthly active users
GA: Google Analytics
MAU: monthly active users
SEO: Search Engine Optimization
SERP: search engine results page
